# Vat Photopolymerization of Sepiolite Fiber and 316L Stainless Steel-Reinforced Alumina with Functionally Graded Structures

**DOI:** 10.3390/ma17122973

**Published:** 2024-06-18

**Authors:** Chang Liu, Hailong Wu, Anfu Guo, Dekun Kong, Zhengyu Zhao, Lu Wang, Lvfa Yin, Guojun Xia, Xiaofei Su, Yingbin Hu

**Affiliations:** 1 School of Mechanical and Automotive Engineering, Liaocheng University, Liaocheng 252000, China; 2 Department of Mechanical and Manufacturing Engineering, Miami University, Oxford, OH 45056, USA

**Keywords:** vat photopolymerization, functionally graded structures, microstructure characterizations, physical properties, mechanical properties

## Abstract

Alumina (Al_2_O_3_) ceramics are widely used in electronics, machinery, healthcare, and other fields due to their excellent hardness and high temperature stability. However, their high brittleness limits further applications, such as artificial ceramic implants and highly flexible protective gear. To address the limitations of single-phase toughening in Al_2_O_3_ ceramics, some researchers have introduced a second phase to enhance these ceramics. However, introducing a single phase still limits the range of performance improvement. Therefore, this study explores the printing of Al_2_O_3_ ceramics by adding two different phases. Additionally, a new gradient printing technique is proposed to overcome the limitations of single material homogeneity, such as uniform performance and the presence of large residual stresses. Unlike traditional vat photopolymerization printing technology, this study stands out by generating green bodies with varying second-phase particle ratios across different layers. This study investigated the effects of different contents of sepiolite fiber (SF) and 316L stainless steel (SS) on various aspects of microstructure, phase composition, physical properties, and mechanical properties of gradient-printed Al_2_O_3_. The experimental results demonstrate that compared to Al_2_O_3_ parts without added SF and 316L SS, the inclusion of these materials can significantly reduce porosity and water absorption, resulting in a denser structure. In addition, the substantial improvements, with an increase of 394.4% in flexural strength and an increase of 316.7% in toughness, of the Al_2_O_3_ components enhanced by incorporating SF and 316L SS have been obtained.

## 1. Introduction

As an indispensable engineering ceramic material, alumina (Al_2_O_3_) ceramics are widely utilized across various sectors, including electronics [1], automotive industries [2], healthcare [3], and other fields. It is commonly used to produce key components such as insulators, capacitors, spark plug electrodes, orthopedic implants, and dental materials. The broad applications are attributed to Al_2_O_3_’s remarkable hardness, stability at elevated temperatures, wear resistance, superior electrical insulation, and other outstanding properties. However, the high intrinsic brittleness of Al_2_O_3_ materials presents challenges to their mechanical strength, impact resistance, and machinability, thus limiting their application effectiveness and reliability when faced with sudden loads and complex processing demands [4]. Facing these challenges, scholars worldwide have introduced second-phase particles like zirconium dioxide [5], carbon nanotubes [6], and LaAlO [7] to improve the toughness performance of Al_2_O_3_ ceramics. Introducing single second-phase particles simplifies the material preparation process, reduces costs, facilitates the uniform distribution of particles within the matrix, and enhances the material’s repeatability and consistency in performance. Despite these benefits, this method has limitations in improving performance, as it cannot optimize multiple properties simultaneously and may not meet the high demands for comprehensive performance required by certain specific applications. Therefore, introducing two different phases can more effectively enhance the multiple properties of Al_2_O_3_ ceramics. Since the homogeneity of a single material may lead to limitations such as restricted performance and the presence of large residual stresses [8], research on functionally graded materials (FGMs) has been gaining significance. The concept of FGMs was initially introduced in the early 1980s [9]. Currently, FGMs are particularly crucial in aerospace and electronic circuit industries, where resistance to high thermal gradients is required [10]. Furthermore, FGMs demonstrate significant potential in the biomedical field, where customized orthopedic implants are needed [11], by mimicking the gradient variation of bones to enhance biocompatibility and biomechanical stability. The primary manufacturing techniques for FGMs include powder metallurgy [12], lamination, chemical vapor deposition [13], physical vapor deposition [14], and additive manufacturing (AM) [15]. Notably, AM offers unparalleled flexibility and precision in the design and production of FGMs, enabling the realization of continuous variations in material properties within a single component and precise control over the distribution of functional gradients. Vat photopolymerization (VPP), as an AM technique, exceeds other AM technologies in terms of dimensional accuracy, temperature stability, and a reduced need for complex support structures [16]. These advantages significantly reduce the production cycle, making producing ceramic components with complex structures and high precision feasible.

To improve the mechanical properties of Al_2_O_3_ ceramics, Wei Zhao et al. [17] explored the toughening of 316L SS Al_2_O_3_-based ceramic FGM using adhesive jet AM, adding specific additives can effectively improve the mechanical properties of Al_2_O_3_ ceramics. Amir Hadian et al. [18] made fiber-reinforced zirconia-toughened Al_2_O_3_ ceramic matrix composites using material extrusion technology, proving that careful control of AM process parameters and material composition can produce high-performance ceramic composites suitable for high-temperature applications. Haidong Wu et al. [19] carried out a study on the preparation of zirconia-reinforced alumina ceramics by stereolithography, and the mechanical properties of the prepared ZTA ceramics were enhanced. Compared to adhesive jet AM and material extrusion-based AM technologies, VPP printing demonstrates higher printing accuracy and better surface quality. Additionally, the above authors have only introduced one phase, while the introduction of two items can combine the advantages of different materials to optimize the performance from multiple aspects. The adoption of gradient printing can overcome the limitations of the homogeneity of a single material, such as non-uniformity of performance and the existence of large residual stresses. Despite these potential benefits, very few scholars have conducted research on VPP of multi-phase particle-reinforced Al_2_O_3_ with functionally graded structures. To fill this gap, this paper comprehensively and systematically studies the effects of varying SF and 316L SS content (2 wt.%, 4 wt.%, 6 wt.%, 8 wt.%) on the performance of Al_2_O_3_ parts produced by VPP printing, including microstructure, phase composition, physical properties, and mechanical performance. In particular, increasing the additive level from 6 wt.% to 8 wt.% did not bring any significant improvement and actually reduced the performance of the parts. Consequently, higher additive levels were not investigated further. SF is a magnesium silicate mineral that is rich in water and known for its distinctive nanoscale fibrous layered structure. This natural resource is valued for its excellent renewable properties, and its utilization has a reversible impact on the environment without causing lasting damage. Additionally, the application of SF usually requires very little energy consumption, which demonstrates its environmental protection and energy-efficiency advantages in various usage scenarios; meanwhile, 316L SS not only has excellent toughness and oxidation resistance [17,20] but also possesses good moldability and economy and is widely used [21], so SF and 316L SS have been selected as the second-phase particles in this study. Different from conventional VPP technology, this study has introduced a novel gradient printing process by creating green bodies with varying densities across various layers. The successful completion of this study provides a methodological reference for developing cost-effective and efficient manufacturing of high-strength and high-toughness Al_2_O_3_, revealing the sintering mechanisms of adding two different types of additives and the performance enhancement mechanisms for Al_2_O_3_ parts. This research also lays a solid theoretical and practical basis for utilizing VPP technology to enhance the strength of Al_2_O_3_ components in industrial applications.

## 2. Experiments and Measurement Procedures

### 2.1. Material Preparation

In this study, we selected α-Al_2_O_3_ powders (models ZY- Al_2_O_3_-4 and ZY- Al_2_O_3_-6, produced by Hebei Chuancheng Metal Materials Co., located in Xingtai, China) with the compositions shown in Table 1. The α-Al_2_O_3_ was selected due to its high hardness, strength, and wear resistance, as well as the outstanding durability and reliability of printed parts. Its excellent electrical insulation and uniform particle size distribution ensure material uniformity and finished product quality. Most importantly, α-Al_2_O_3_ powders offer a good balance of performance and cost. Magnesium oxide (from Hebei Badu Metal Materials Co., Ltd., Xingtai, China) was used as a sintering aid. The study used spherical 316L SS powder with a particle size of 15–53 μm (Farsoon Technologies Co., LTD, Changsha, China). The UV-curable resin consisted of 1,6-hexanediol diacrylate (HDDA, Sicheng Optoelectronic Materials Co., Ltd., Chengdu, China), trimethylolpropane triacrylate (TMPTA, Sicheng Optoelectronic Materials Co., Ltd., Chengdu, China), and epoxy resin (E51, Sicheng Optoelectronic Materials Co., Ltd., Chengdu, China). The photopolymerization reaction of the UV resin was initiated using a photoinitiator (1173, Sicheng Optoelectronic Materials Co., Ltd., Chengdu, China). To mitigate the adverse effects of particles on the performance of the ceramic slurry and to avoid printing issues caused by overly large particles or the tendency of overly small particles to agglomerate and be difficult to disperse, this study chose to use 600 mesh SF and 316L SS particles with a size range of 15–53 μm for experimental research.

**Figure 1:**
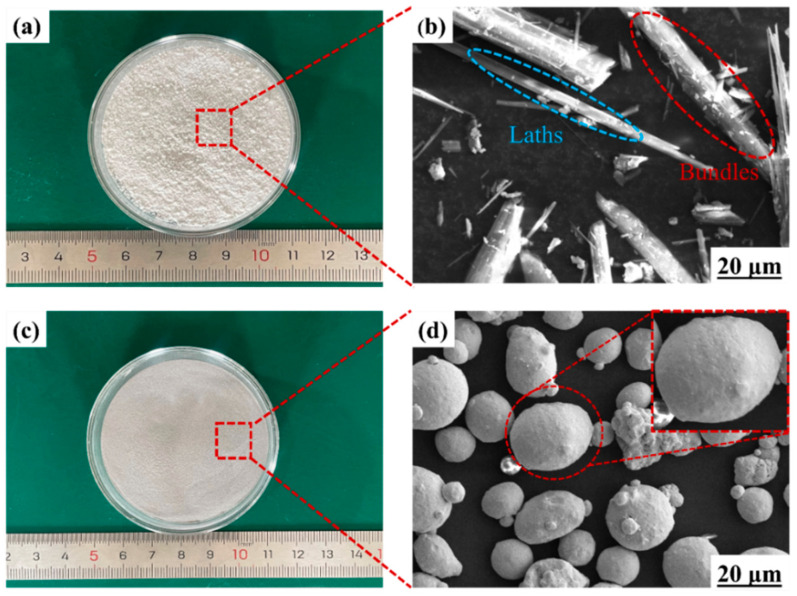
(**a**) A picture and (**b**) an SEM image of SFs; (**c**) a picture and (**d**) an SEM image of 316L SS. Macroscopically, the SFs are white powders, while microscopically they are in forms of bundles and laths. The length of SFs ranges from 20 to 60 µm.

Figure 1a,b depict a picture and SEM image of SFs, respectively. On a macroscopic scale, SFs appear as a white powder. The length of SFs mainly ranges between 20 and 60 µm, with an average length of 51.68 µm. The diameter of the SFs varies from 0.3 to 1.5 µm, with an average diameter of 1.01 µm. Figure 1c,d depict a picture and SEM image of 316L SS, respectively. Macroscopically, 316L SS appears as black particles. Under the microscope, 316L SS particles are spherical, with diameters mainly concentrated in the 15–53 μm range. According to the manufacturer’s material specifications, the powder material’s chemical composition is shown in Table 2.

### 2.2. Preparation of Toughened Al_2_O_3_ Ceramics with SFs and 316L SS

The preparation process for Al_2_O_3_ ceramics primarily encompasses three key steps: first, the formulation of Al_2_O_3_ slurry; second, printing using VPP technology; and finally, post-processing of the printed Al_2_O_3_ ceramics.

**Figure 2:**
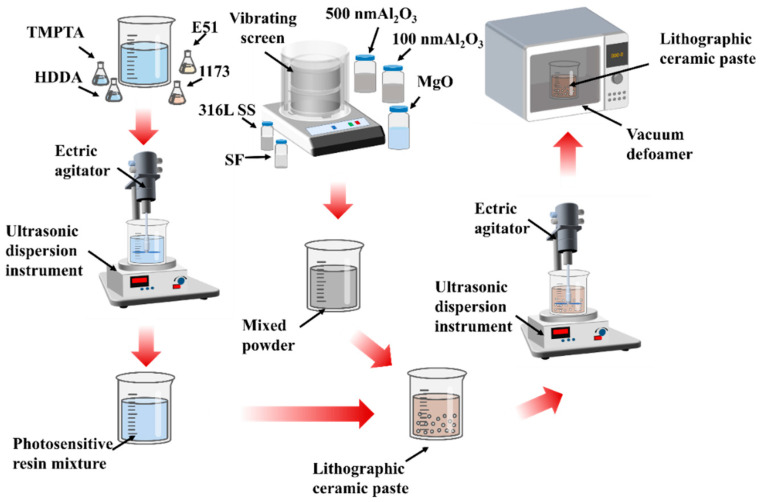
A diagram showing a schematic of the Al_2_O_3_ slurry preparation.

Figure 2 illustrates the preparation process flowchart for Al_2_O_3_ slurry. Initially, a vibratory sieve (500 μm, Xinxiang Qiaomei Machinery Co, Ltd., Xinxiang, China) is utilized to pre-treat Al_2_O_3_ powders of 500 nm and 100 nm, and magnesium oxide powder of 200 nm to prevent powder agglomeration. Subsequently, these powders are mixed in a mass ratio of 55.2:13.8:1. During the preparation of the photosensitive resin, HDD, trimethylolpropane TMPTA, and E51 are blended in a 1:1:1 mass ratio, with the addition of 1 wt.% 1173.

The blend was dispersed with the help of an ultrasonic disperser (P-010 T, Shenzhen United Cleaning Equipment Co., Ltd., Shenzhen, China) and agitated using an electric mixer (JJ-160 W, Xicheng Xinrui Instrument Factory, Changzhou, China) for 20 min to achieve homogeneous mixing. Next, the formulated powder was mixed with the photosensitive resin at a mass ratio of 7:3, SF and 316L SS were added according to the experimental design, and the mixture was thoroughly mixed using an ultrasonic disperser and an electric mixer.

**Figure 3:**
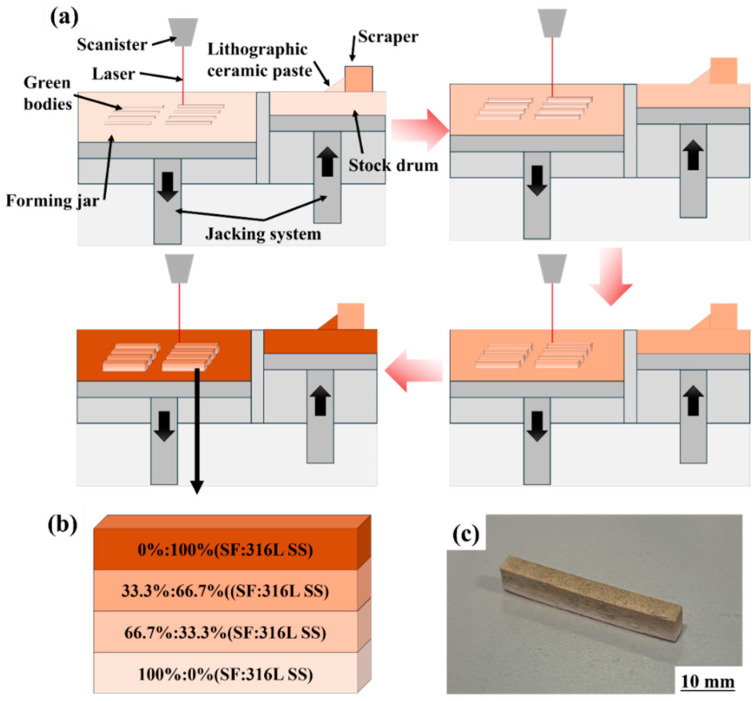
(**a**) Gradient printing process flow diagram, (**b**) Al_2_O_3_ green body printing diagram, and (**c**) printed physical image.

In this study, the weight ratios of the additives were set to 2 wt.%, 4 wt.%, 6 wt.%, and 8 wt.%, respectively; the green body was divided into four levels of concentration for printing, and the printing process flow is shown in Figure 3a; the ratios of SF to 316L SS were, in descending order, 100%: 0%, 66.7%: 33.3%, 33.3%: 66.7% and 0%. 100%, while the layers with SF to 316L SS ratios of 100%:0%, 66.7%:33.3%, 33.3%:66.7%, and 0%:100% are named S1, S2, S3, and S4, respectively, for ease of expression, and the schematic and physical drawings of the Al_2_O_3_ ceramics are shown in Figure 3b and 3c, respectively.

**Figure 4:**
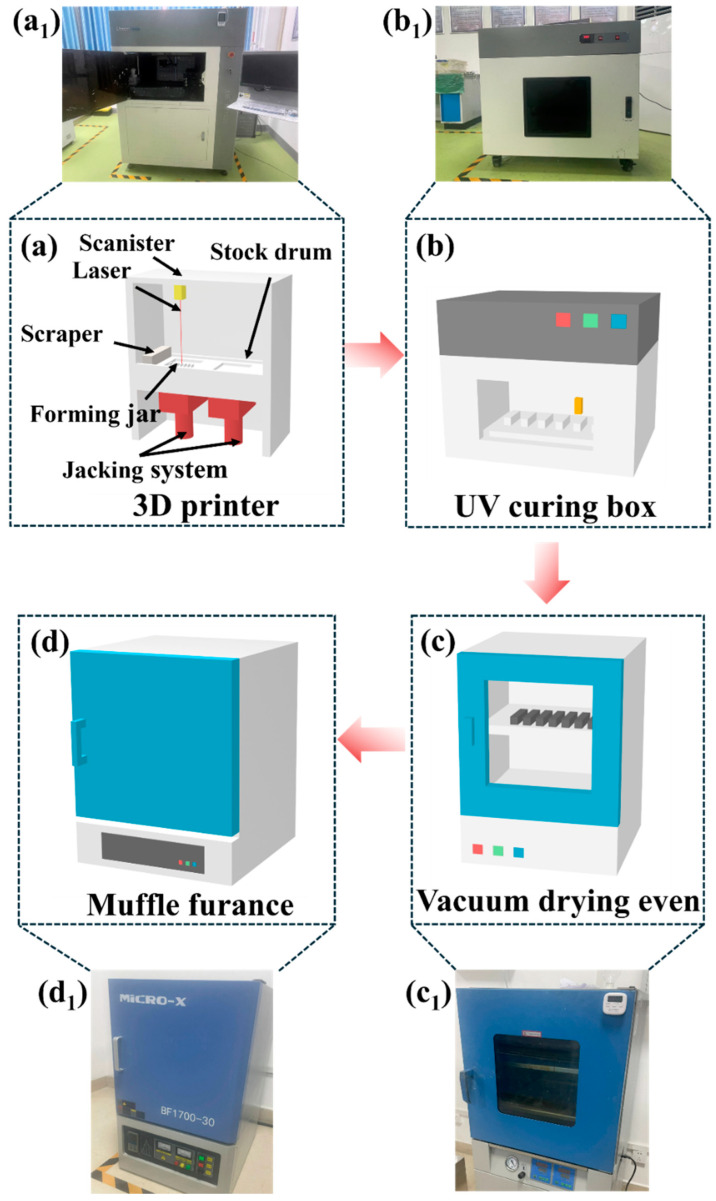
Diagrams showing schematics and objects of (**a**) VPP printing of Al_2_O_3_ green bodies, (**b**) post-curing of Al_2_O_3_ green bodies in a UV curing box, (**c**) drying of Al_2_O_3_ green bodies in a vacuum drying oven, and (**d**) degreasing and sintering processes. (**a**_1_–**d**_1_) The images of the corresponding equipment.

Al_2_O_3_ green bodies with dimensions of 3 × 4 × 30 mm were printed using a VPP printer (AME RP150, Shanghai United Technology Co., Ltd., Shanghai, China), which was equipped with a laser of 355 nm wavelength, as shown in Figure 4a. During the printing process, the Al_2_O_3_ slurry was first placed in the feed cylinder, and a scraper transferred the slurry from the feed barrel to the forming barrel, creating a thin layer of material. The laser then solidified parts of the slurry according to a preset pattern. Subsequently, the feed barrel and the printing barrel moved up and down synchronously, with the forming barrel descending by 0.05 mm each time. To prevent slurry leakage from the sides, resulting in an insufficient layer thickness of 0.05 mm in the forming barrel, the lifting height of the feed barrel was set to 0.06 mm. After completing the S1 layer, the ceramic slurry in the feed cylinder and forming barrel was replaced to continue printing the S2 layer. This step was repeated until a complete green body was formed.

After printing, to remove any uncured resin on the surface of the green bodies, they were first carefully wiped with alcohol wipes. This step aimed to prevent the uncured resin from adversely affecting the subsequent curing process. Following this, the green bodies were placed in a UV curing box (PCU 80, Shanghai VPP Technology Co., Ltd., Shanghai, China) for an additional curing treatment lasting 15 min, as shown in Figure 4b. After the UV curing treatment, the green bodies were transferred to a vacuum-drying oven for drying, ensuring the complete removal of any residual moisture, as depicted in Figure 4c.

The post-processing flow of the green bodies involves two key steps: debinding and sintering, which were carried out in a muffle furnace (BR-17 M, Bona Thermal Furnace Co., Ltd., Zhengzhou, China), as shown in Figure 4d.

**Figure 5:**
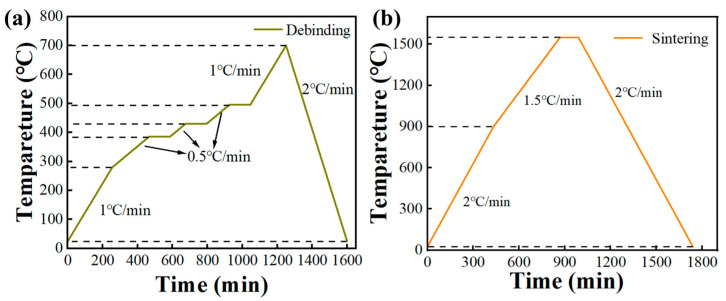
Thermal analysis tests. (**a**) Debinding temperature curve. (**b**) Sintering.

Based on previous experimental data [22], the critical temperatures for debinding were determined to be 275 °C, 385 °C, 430 °C, and 495 °C. Figure 5a displays the entire debinding temperature curve over time. During debinding, a relatively low heating rate (0.5–2 °C/min) is employed to prevent cracking and deformation of the green bodies, with set holding times to ensure the complete evaporation of organic substances. The removal section undergoes sintering at elevated temperatures to achieve consistent density and uniformity of the Al_2_O_3_ components. Figure 5b illustrates the sintering temperature curve. The sintering process involves heating the samples to 900 °C (2 °C/min), then raising the temperature to 1500 °C (1.5 °C/min) and holding them at this temperature for two hours to complete sintering. Finally, the samples are cooled to room temperature at 2 °C/min to prevent cracking. A series of steps are meticulously followed to ensure the quality and performance of the final product.

### 2.3. Characterization

The study utilized a high-precision DualBeam Focused Ion Beam Scanning Electron Microscope (model GX4, Thermo Fisher Scientific, Waltham, MA, USA) to conduct in-depth observations of the surface and microstructure of the samples post-sintering. This FIB-SEM equipment was geared up with an energy dispersive spectroscopy (EDS) system, which was used for detailed assessment of the elemental distribution and fabric composition, providing more accurate information on the microstructure.

Phase analysis of the crystalline materials was completed using X-ray diffraction (XRD) technology, with the instrument being a D/max-2200PC XRD (Rigaku Corporation, Tokyo, Japan). The testing angle of this XRD was set between 20° to 80°, with a scanning speed of 5°/min. X-ray photoelectron spectroscopy (XPS) analysis was performed using an Escalab Xi+ X-ray Photoelectron Spectrometer (Thermo Fisher Scientific, Waltham, MA, USA).

After the debinding and sintering treatments, organic components in the green bodies were thoroughly removed, and concurrently, the growth of ceramic particles filled the original voids, reducing their volume. To calculate the volumetric shrinkage ratio during the sintering process, this study measured the dimensions of the green bodies before and after the debinding and sintering treatments along the X, Y, and Z dimensions.

(1) $$C=\frac{L_{1}-L_{2}}{L_{1}}\times 100\%$$

Herein, *C* represents the percentage of shrinkage in the sintered samples. Before the debinding process, *L*_1_ represents the dimension (mm) of the green body, while *L*_2_ denotes the dimension (mm) of the sample in the same direction after sintering.

Using the specific gravity determination method, volume density, apparent porosity, water absorption, and relative density were measured and calculated using an automatic electronic densimeter (model Byes-300B, Bang Precision Instruments Co., Ltd., Shanghai, China) with the following formulas.

(2) $$D_{b}=\frac{G_{1}\times D_{W}}{G_{1}-G_{3}}\times 100\%$$

(3) $$O=\frac{G_{2}-G_{1}}{G_{2}-G_{3}}\times 100\%$$

(4) $$W=\frac{G_{2}-G_{1}}{G_{1}}\times 100\%$$

where the symbol “*D*_b_” denotes the volumetric density of the sintered samples (g/cm^3^), O represents the surface porosity of the sintered samples (%), *W* is the water absorption rate of the sintered samples (%), *D_w_* is the density of water (g/cm^3^), *G*_1_ is the mass of the dry sample (g), *G*_2_ is the wet mass of the sample (g), and *G*_3_ is the buoyant mass of the sample (g).

The three-point bending tests conducted in this study strictly adhered to the GB/T6569-2006 standard [23], utilizing a microcomputer-controlled electronic universal testing machine (JD3310W, Hebei Cangzhou Yixuan Experimental Instrument Co., Ltd., Cangzhou, China). During the tests, the loading speed was set at 0.05 mm/min, with a chosen span of 20 mm. To ensure high consistency and reliability of the test results, five samples were carefully tested in each test category. The bending strength and strain values of the samples were then calculated using the following equations [22].

(5) $$\sigma =\frac{3FH}{2{\mathrm{bd}}^{2}}$$

(6) $$\varepsilon =\frac{6D_{f}d}{H^{2}}$$

The symbols in the equation represent the following variables: σ represents flexural strength (MPa), *F* stands for the maximum load (N), L denotes the distance between the lower fixtures (mm), b is the width of the sample (mm), d represents the height of the sample (mm), ε signifies the strain at flexural strength (%), and *D_f_* stands for the displacement at the midpoint (mm).

Toughness is a characteristic that measures how well a material can withstand the propagation of cracks. It represents the maximum stress intensity factor a material can withstand under static load before fracturing begins from a region containing sharp cracks. The calculation can be performed using the following formula [24]:

(7) $$K_{IC}=\frac{3PH}{2{\mathrm{bd}}^{1.5}}\times {\left(\frac{a}{d}\right)}^{0.5}\times \frac{1.99-\frac{a}{d}\times \left(1-\frac{a}{d}\right)\left(2.15-3.93\frac{a}{d}+2.7{\left(\frac{a}{d}\right)}^{2}\right)}{\left(1+2\frac{a}{d}\right){\left(1-\frac{a}{d}\right)}^{1.5}}$$

In the formula, *K*_IC_ represents the fracture toughness (MPa/m), and a is the crack length from the specimen surface to the crack tip (mm).

## 3. Results and Discussion

### 3.1. Debinding and Sintering of VPP-Printed Green Bodies

**Figure 6:**
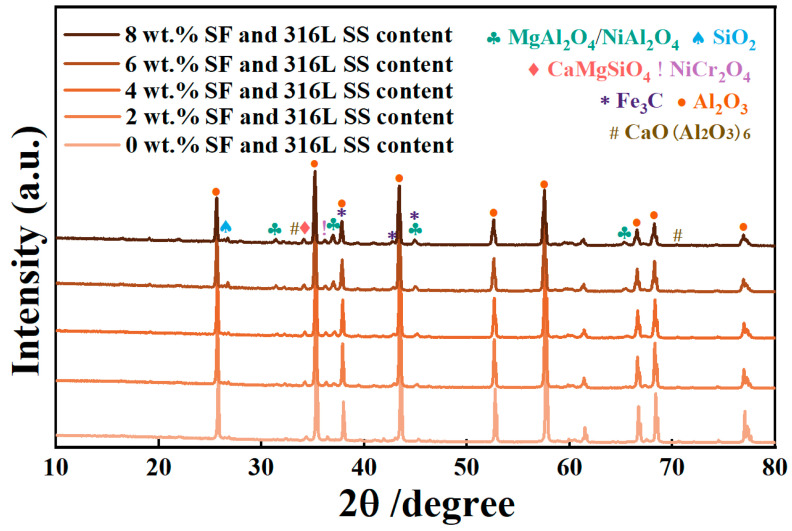
Effects of SF and 316L SS content on XRD.

This study further analyzed the phase composition of sintered Al_2_O_3_ components using XRD technology. As shown in Figure 6, the undoped Al_2_O_3_ samples exhibited distinct diffraction peaks, corresponding uniquely to the alpha phase of α-Al_2_O_3_, confirming that the α-phase is the primary component of the Al_2_O_3_ sample structure. For Al_2_O_3_ parts doped with SF and 316L SS, the emergence of additional diffraction peaks indicates a more complex phase composition of the samples. Introducing these additives promoted the formation of secondary phases such as Fe_3_C, NiAl_2_O_4_, and NiCr_2_O_4_, reflecting the presence of iron, nickel, and chromium elements from 316L SS. Furthermore, the formation of phases such as CaO(Al_2_O_3_)_6_, MgAl_2_O_4_, CaMgSiO_4_, and SiO_2_ revealed complex interactions occurring during the sintering process and reflected the presence of Si and Mg elements from SF. The intensity of their diffraction peaks is relatively weak due to the relatively low content of SF and 316L SS in the mixed powder.

The crystallite size of Al_2_O_3_ can be determined using the Scherrer equation based on the XRD spectrum [25].

(8) $$d=\frac{K\lambda }{B\cos\theta }$$

The formula signifies that “*d*” stands for the average crystallite size of Al_2_O_3_; “*K*” represents the Scherrer constant; “*λ*” indicates the wavelength of the X-rays; “*B*” is the full width at half maximum (FWHM) of the Al_2_O_3_ peak; and “*θ*” denotes the angle of incidence of the X-rays.

An increase in SF and 316L SS resulted in a decrease in the intensity of the diffraction peaks of the Al_2_O_3_ sample, while the full width at half maximum (*B*) increased from 0.1631 to 0.1642. According to the Scherrer equation, an increase in peak width indicates a reduction in the size of Al_2_O_3_ crystallites. This reduction in crystallite size can be attributed to the presence of CaO and MgO, which react with Al_2_O_3_ to form CaO(Al_2_O_3_)_6_ and MgAl_2_O_4_. These phases inhibit the diffusion of Al_2_O_3_ grains, preventing excessive growth at high temperatures and thus achieving a refinement of the grains [26,27,28,29]. X-ray diffraction analysis shows that at some point in the sintering process, CaO produced from SFs reacts with Al_2_O_3_ to generate CaO(Al_2_O_3_)_6,_ which can inhibit the formation of a glassy phase, preventing abnormal grain growth [22], thus improving the flexural strength of Al_2_O_3_ parts [30]; meanwhile, 316L SS generates NiO and NiCr_2_O_4_. NiO reacts with Al_2_O_3_ to form NiAl_2_O_4_, NiAl_2_O_4,_ and NiCr_2_O_4_, when present as a secondary phase in a ceramic matrix, which can effectively limit crack propagation by bridging or deflecting the crack path, thus enhancing the material’s toughness [31,32,33,34].

**Figure 7:**
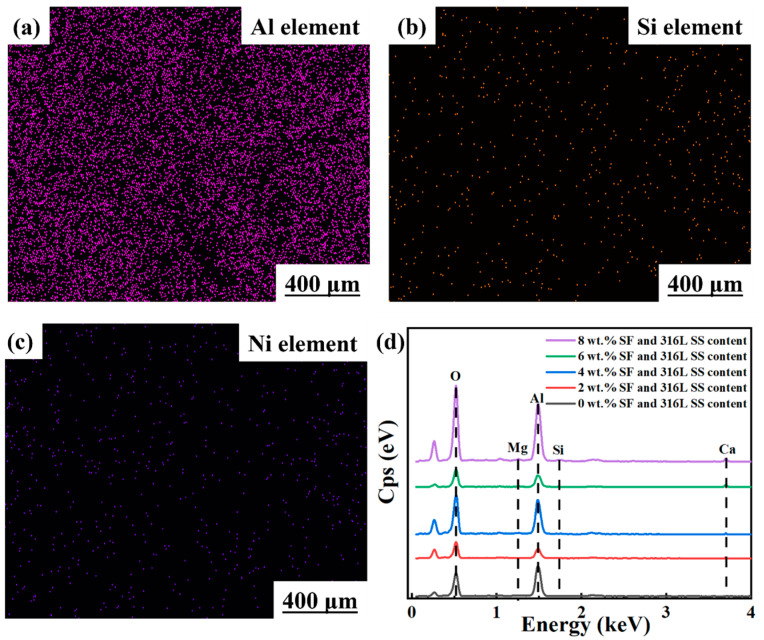
The EDS elemental distribution maps of (**a**) Al element, (**b**) Si element, and (**c**) Ni element. Of an Al_2_O_3_ sample with 6 wt.% SF and 316L SS content. (**d**) An EDS energy spectrum diagram.

To further verify the distribution of SF and 316L SS in Al_2_O_3_ samples, an energy-dispersive X-ray spectroscopy (EDS mapping) analysis was applied to Al_2_O_3_ samples containing 6 wt.% SF and 316L SS. The analytical results are presented in Figure 7. In this analysis, the element Al was used to represent Al_2_O_3_, Si for SF, and Ni to denote 316L SS. Observations from Figure 7b revealed a random distribution of Si elements within the sample without any fibrous structures, indicating that SF had completely melted and transformed into a glass phase during the sintering process [22]. Furthermore, the random distribution of Ni elements shown in Figure 7c confirmed that 316L SS particles had also completely melted and were uniformly dispersed in the Al_2_O_3_ matrix ceramic in a liquid form during sintering [17], demonstrating the important role of SF and 316L SS in connecting Al_2_O_3_ grains and promoting part densification [17,22]. Figure 7d displays the energy spectra of Al_2_O_3_ samples. Characteristic peaks at 1.25 keV, 1.73 keV, and 3.70 keV correspond to the characteristic X-ray emission peaks of Mg, Si, and Ca, respectively. Due to the low addition levels of 316L SS, its specific peaks were not visually observed in the energy spectrum of the Al_2_O_3_ samples. However, the main elements of 316L SS could still be identified through related experiments. Additionally, with the increase in SF content, a gradual increase in the relative content of each target element was observed compared to samples without added SF, thus confirming that these elements are among the main components of SF.

**Figure 8:**
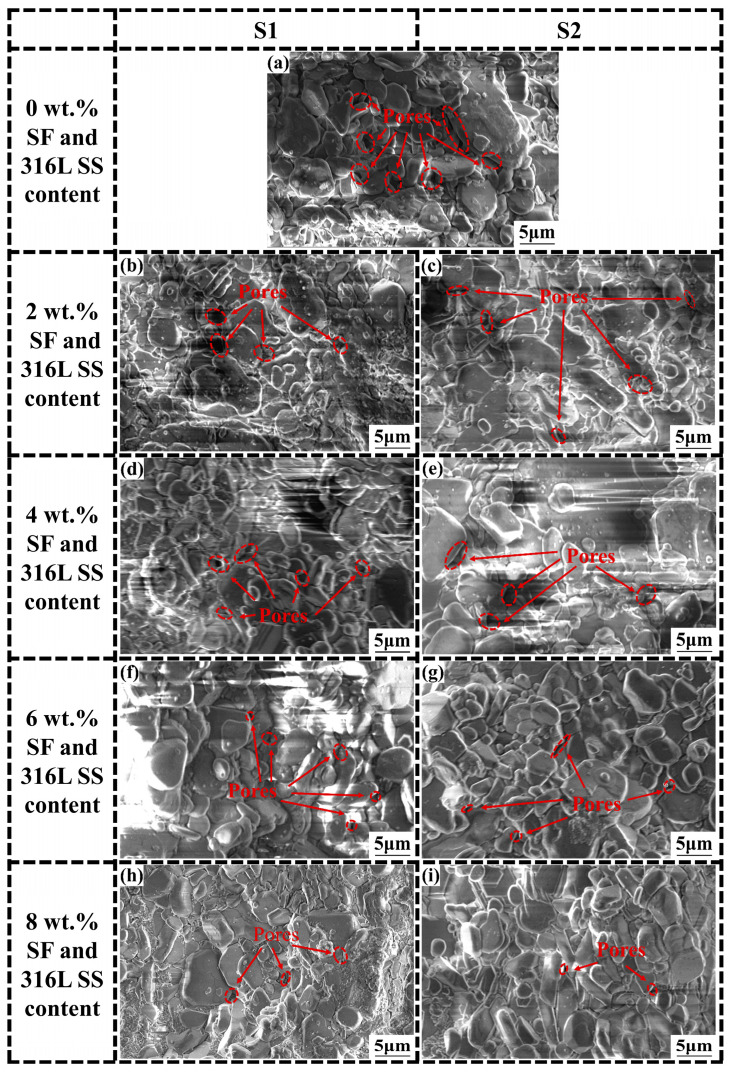
SEM images of sintered samples with the addition of (**a**) 0 wt.%, (**b**,**c**) 2 wt.%, (**d**,**e**) 4 wt.%, (**f**,**g**) 6 wt.%, and (**h**,**i**) 8 wt.% SF and 316L SS addition for S1 and S2.

**Figure 9:**
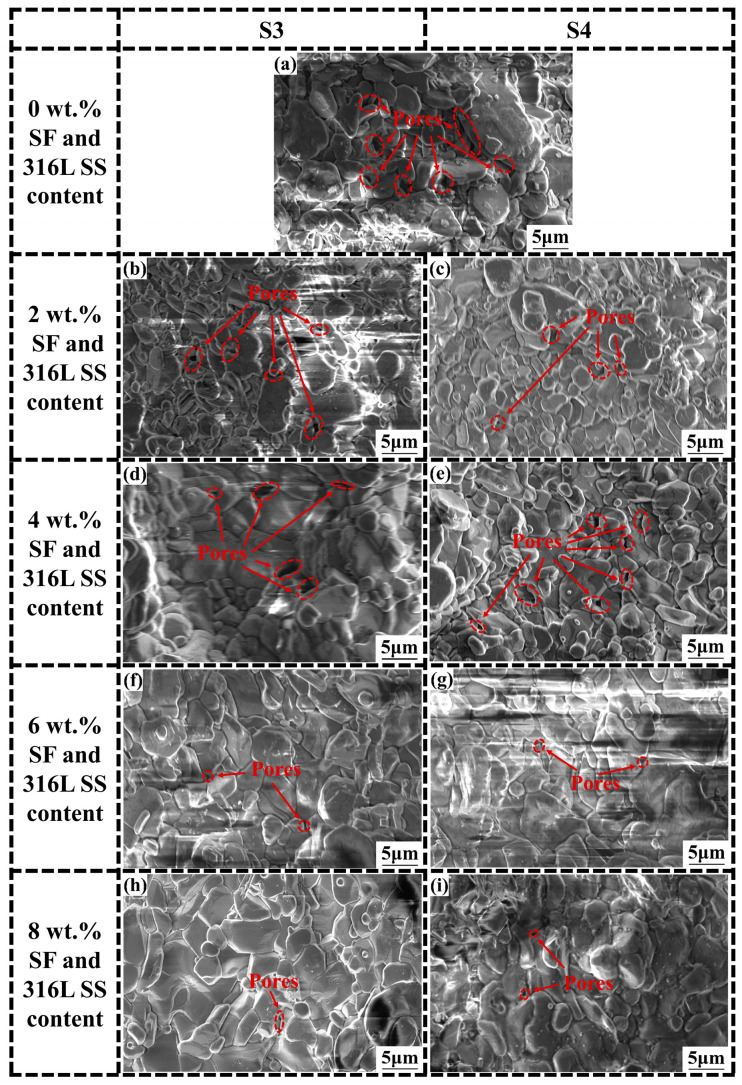
SEM images of sintered samples with the addition of (**a**) 0 wt.%, and (**b**,**c**) 2 wt.%, (**d**,**e**) 4 wt.%, (**f**,**g**) 6 wt.%, and (**h**,**i**) 8 wt.% SF and 316L SS addition for S3 and S4.

**Figure 10:**
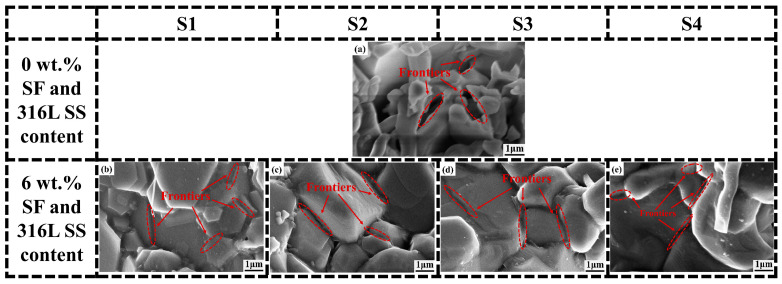
SEM images of sintered samples with the addition of (**a**) 0 wt.% and 6 wt.% SF and 316L SS addition for (**b**) S1, (**c**) S2, (**d**) S3, and (**e**) S4.

To achieve a higher grasp of the microstructure of the samples, SEM imaging analyses were conducted on Al_2_O_3_ samples of S1, S2, S3, and S4, which contain varying contents of SF and 316L SS at concentrations of 0 wt.%, 2 wt.%, 4 wt.%, 6 wt.%, and 8 wt.%. The accelerating voltage used in the experiment is 10kV, and the magnification is 10,000 times. SEM images for S1 and S2 are shown in Figure 8 and for S3 and S4 in Figure 9. After careful analysis of the SEM images from S1 to S4; it was observed that as the SF and 316L SS content gradually increased from 0 wt.% to 8 wt.%, significant changes in the microstructure of the material were evident. In the absence of SF and 316L SS, the Al_2_O_3_ samples exhibited a higher porosity and larger pore sizes. These pores, being spaces within the material, weaken its mechanical strength and durability. However, as the addition levels of SF and 316L SS increased, a significant reduction in porosity and decrease in pore size was observed, particularly in samples with 6 wt.% and 8 wt.% additions of SF and 316L SS, where pores were almost invisible in SEM images. This indicates that SF and 316L SS melted and filled these spaces at the high temperature of 1500 °C, thereby promoting material densification and forming a uniformly dense structure [17,22]. At the same time, we carried out higher-magnification SEM imaging analysis of Al_2_O_3_ ceramics with 0 wt.% and 6 wt.% SF and 316L SS additions, as shown in Figure 10, where it can be seen that the grains are more closely packed with each other, thus further confirming the above conclusion. Furthermore, it was observed that samples with a 6 wt.% addition exhibited minor signs of aggregation. This phenomenon became more pronounced in samples with an 8 wt.% addition, suggesting that excessive amounts of SF and 316L SS could lead to aggregation. Overall, the addition of SF and 316L SS significantly optimized the microstructure of the Al_2_O_3_ samples, resulting in a more densified structure.

**Figure 11:**
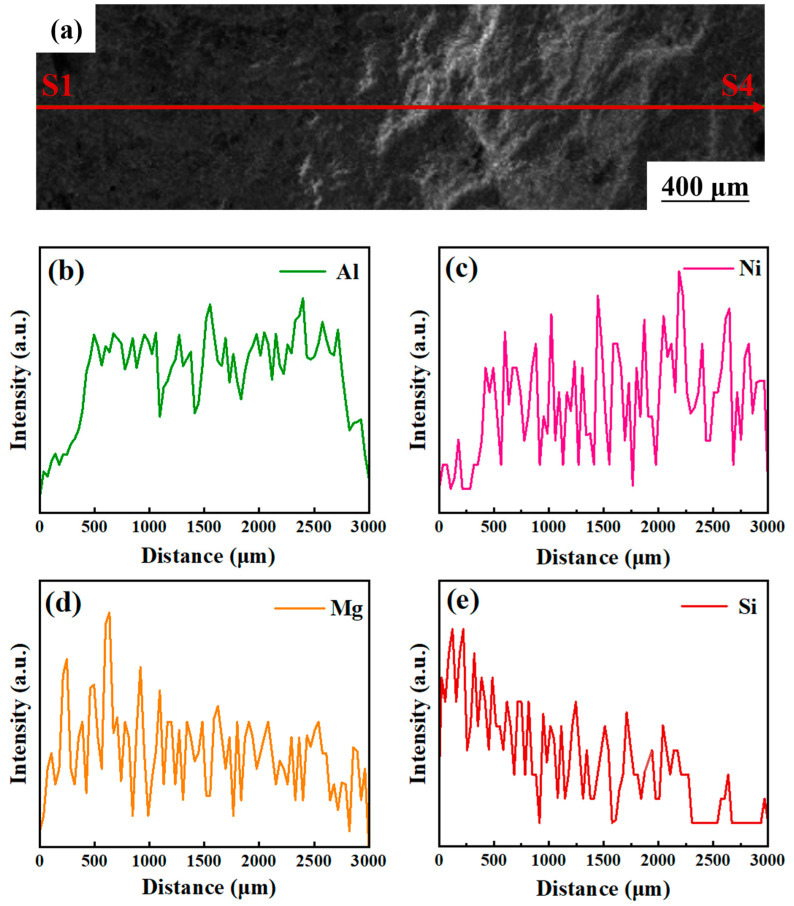
The line scanning images of the Al_2_O_3_ gradient sample: (**a**) microstructure, (**b**) Al elements, (**c**) Ni elements, (**d**) Mg elements, and (**e**) Si elements.

Figure 11 displays the line scanning results for the Al_2_O_3_ samples with 6 wt.% SF and 316L SS added, scanning from S1 to S4. These paths are marked with a bold red baseline in Figure 11a. As seen in Figure 11a, localized porosity is present in the interface organization, but continuous structural cracks were not observed. This indicates that effective connections have been formed between the layers within the gradient samples, proving the rationality of the debinding and sintering process parameters.

In Figure 11b, the distribution density and peak intensity of aluminum (Al) elements within the Al_2_O_3_ matrix are observed to maintain consistency across the entire interface. Figure 11c reveals the distribution of nickel (Ni) elements in 316L SS, where the distribution density and peak intensity on the right side are significantly higher than on the left, aligning with the increasing trend of 316L SS in the composition of various Al_2_O_3_ samples. Furthermore, as shown in Figure 11d,e, the distribution density and peak intensity of magnesium (Mg) and silicon (Si) elements from SF are significantly higher on the left side than on the right, consistent with the trend of SF in the composition of Al_2_O_3_ samples.

**Figure 12:**
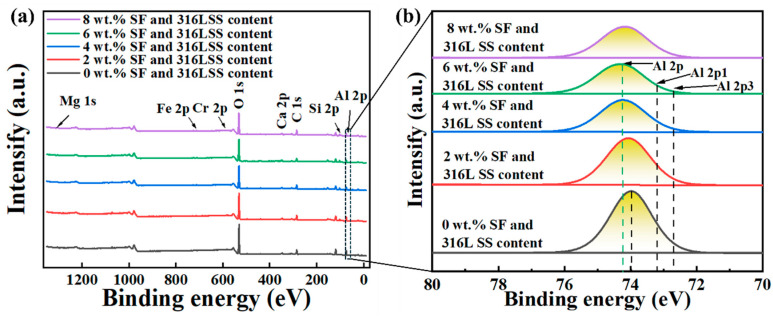
Effects of SF and 316L SS content on (**a**) XPS full spectrum, (**b**) XPS fine spectrum of Al 2p.

XPS was employed to characterize the chemical composition of sintered Al_2_O_3_ samples, with the results shown in Figure 12a. Elements such as magnesium (Mg), iron (Fe), chromium (Cr), calcium (Ca), silicon (Si), carbon (C), and oxygen (O) were detected. The absence of detectable nickel (Ni) may be attributed to its low content, though XRD experimental results indicate its presence in the Al_2_O_3_ samples’ chemical composition. Two factors can explain the occurrence of carbon: first, the sample is exposed to environments with carbon during storage, transport, and analysis, and second, the XPS surface analysis is highly sensitive in detecting carbon peaks. With an increase in the total addition of SF and 316L SS, peaks at 100.1 eV, 346.6 eV, and between 1303.3 eV, 710 eV, and 574 eV become more pronounced, consistent with XRD and EDS results. Figure 12b shows a very broad spectral peak for Al 2p, attributed to the minimal energy difference (0.44 eV) between spin-orbit split peaks Al 2p3 and Al 2p1, which is beneath the energy resolution of XPS. Thus, a single broad peak is observed and fitted in Figure 12a.

In Figure 12b, it is also observed that the XPS peak shifts to the left with an increase in the total addition of SF and 316L SS. However, when the addition increases from 6 wt.% to 8 wt.%, the peak position hardly moves, indicating that the addition of SF and 316L SS at 6 wt.% enhances the binding energy of the Al element, improving the binding stability of Al_2_O_3_ and achieving optimal effects; further addition beyond this does not result in additional performance improvements.

### 3.2. Effects on Physical Properties

The shrinkage rate of sintered Al_2_O_3_ ceramics is a crucial factor affecting the precision of ceramic components. During the sintering process, the linear shrinkage rate reflects the degree of material densification [35,36], a process crucial for ensuring the dimensional accuracy of the final product. This issue is particularly pronounced in 3D-printed ceramic components produced using layer-by-layer printing methods due to the unevenness of internal binding forces. The unevenness of these binding forces leads to anisotropic shrinkage during the sintering process. This phenomenon is especially evident in complex structural ceramic core components with high demands for dimensional accuracy, resulting in unacceptable deformation and irrational shrinkage rates in the final product.

**Figure 13:**
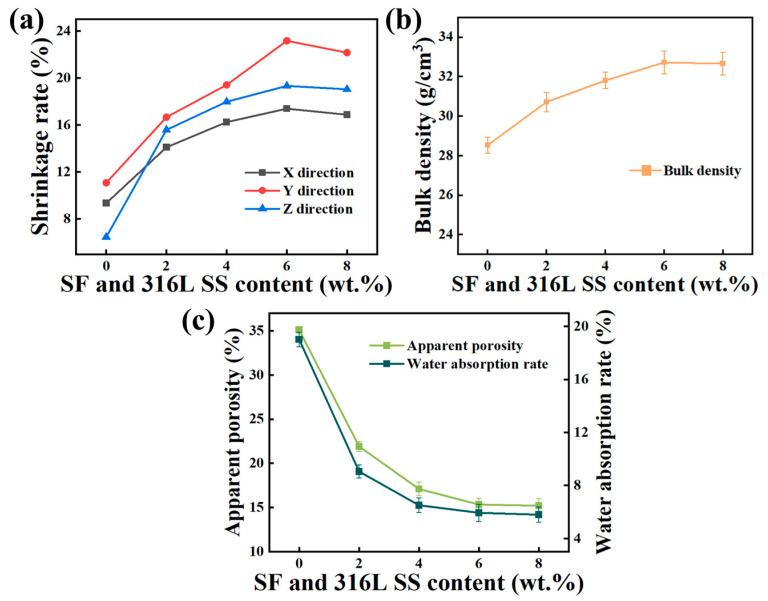
Effects of SF and 316L SS contents on (**a**) shrinkage rates along X, Y, and Z directions, (**b**) bulk density, and (**c**) apparent porosity and water absorption rates of Al_2_O_3_ samples.

The elimination of photosensitive resin and the subsequent densification procedure are primary causes of shrinkage observed in ceramics post-sintering in the VPP printing process. The photosensitive resin used as a binder during VPP printing is completely removed prior to sintering, with densification subsequently achieved through the sintering process. During this process, internal porosities in the material are progressively eliminated, leading to volume shrinkage. As illustrated in Figure 13a, the shrinkage rate of Al_2_O_3_ ceramics increases in all directions as the content of SF and 316L SS rises from 0 wt.% to 6 wt.%. However, a slight decrease in the shrinkage rate in all directions is observed as the content increases from 6 wt.% to 8 wt.%. From a thermodynamic perspective, the increase in SF and 316L SS content from 0 wt.% to 6 wt.% effectively enhances the system’s surface energy, facilitating wetting and diffusion between particles, increasing contact and bonding among particles, and enhancing the driving force for sintering. This promotes the densification process of Al_2_O_3_, thereby expanding the shrinkage rate [19]. When the content of SF and 316L SS increases from 6 wt.% to 8 wt.%, reaching their threshold, excessive addition of SF and 316L SS leads to aggregation. Areas of agglomeration exhibit lower reactivity and, thus, are less likely to densify. Additionally, the formation of an excessive liquid phase can cause abnormal grain growth, creating defects that inhibit densification, resulting in a reduction in shrinkage rate [37]. This aligns with the results observed in the SEM images.

During the observation of the sintering process of ceramic parts, it was noted that the shrinkage rate in the Y direction was more significant compared to the X and Z directions. The pronounced shrinkage in the Y direction can be attributed to its representation of the printing direction, where the ceramic components are constructed through layer-by-layer curing. In this process, each newly formed, not fully cured layer partially bonds with the layer below it, inevitably leading to the formation of gaps between adjacent layers. These gaps serve as the primary pathways for the organic resin to evaporate during the subsequent debonding process. As the debonding process completes and the temperature further increases, these gaps initially expand and then gradually close during the sintering process, increasing the material’s density.

Figure 13b illustrates the impact of SF and 316L SS content on volumetric density. As the content of SF and 316L SS increases from 0 wt.% to 6 wt.%, there is a slight increase in volumetric density, while an increase from 6 wt.% to 8 wt.% results in a slight decrease, aligning with the analysis and results of its shrinkage rate.

The consequences of SF and 316L SS content material on apparent porosity and water absorption are proven in Figure 13c. As the content of SF and 316L SS increases from 0 wt.% to 6 wt.%, there is a rapid decrease in both porosity and water absorption rates. However, when the content increases from 6 wt.% to 8 wt.%, the reduction in porosity and water absorption rates is only slight and not significant.

The vast reduction in porosity and water absorption rates as the content of SF and 316L SS increases from 0 wt.% to 6 wt.% is attributed to SF and 316L SS melting into a liquid section and filling the pores, greatly reducing the porosity and water absorption rates of the Al_2_O_3_ components [17,22]. However, the decrease in porosity and water absorption rates was not significant when the content of SF and 316L SS increased from 6 wt.% to 8 wt.%. This is attributed to the difficulty in achieving uniform dispersion of SF and 316L SS in the Al_2_O_3_ matrix at excessive levels, where the interaction between filler particles leads to aggregation, forming micro-scale heterogeneous regions. This agglomeration restricts the further reduction of porosity and water absorption rates [38]. A decrease in porosity is beneficial for the enhancement of mechanical properties, per the Rysweitsch equation [39].

(9) $$\sigma =\sigma_{0}\exp(-\alpha p)$$

In the equation, σ represents flexural strength in MPa; σ_0_ is flexural strength when the porosity is 0 (in MPa); *p* is porosity (%); and α is a constant. This equation shows that the flexural strength of ceramics increases as the porosity decreases.

In summary, adding an appropriate amount of SF and 316L SS can effectively improve shrinkage rate and relative density and reduce the porosity of Al_2_O_3_ ceramics. However, the excessive addition of SF and 316L SS does not significantly enhance these properties and may even diminish performance.

### 3.3. Effects on Mechanical Properties

**Figure 14:**
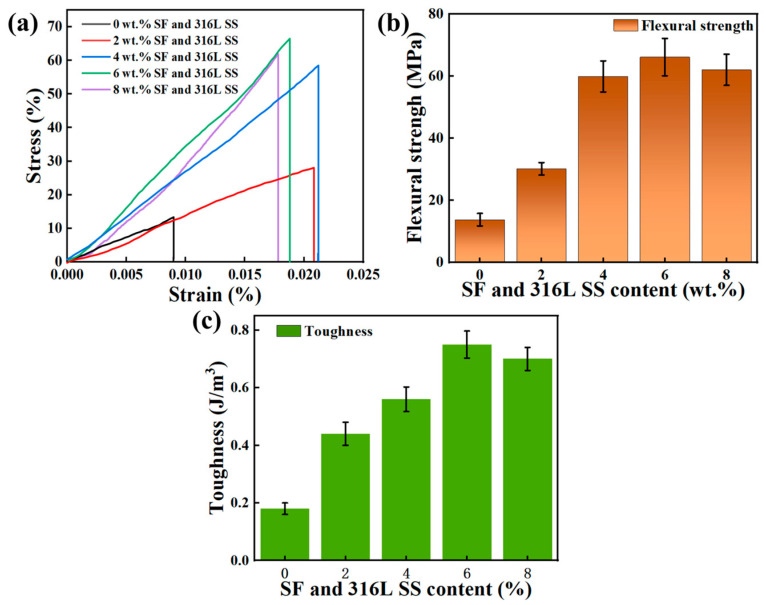
Mechanical properties of Al_2_O_3_ samples with different SF and 316L SS contents: (**a**) Stress–strain curves, (**b**) flexural strength, and (**c**) toughness.

Figure 14a displays the stress–strain curves of Al_2_O_3_ specimens with varying contents of SF and 316L SS. Before fracture, all specimens exhibit a linear increase in stress with increasing strain, indicating that elastic deformation predominates over plastic deformation during the bending tests. The flexural strength is illustrated in Figure 14b, showing that specimens with a 6 wt.% addition of SF and 316L SS exhibit higher flexural strength and greater strain compared to Al_2_O_3_ specimens without added SF and 316L SS. This enhancement is primarily attributed to the liquid phase formed by SF and 316L SS, which fills voids and bonds Al_2_O_3_ particles together. Furthermore, as the SF and 316L SS content increases from 0 wt.% to 4 wt.%, there is a significant increase in maximum strain and flexural strength. This can be explained by the increased formation of the liquid phase with higher SF and 316L SS content, leading to a reduction in gaps and voids within the part, thus improving mechanical performance [17,22]. However, increasing the SF and 316L SS content from 4 wt.% to 6 wt.% results in only a slight increase in flexural strength, with a decrease in maximum strain, and further increasing the content from 6 wt.% to 8 wt.% leads to a reduction in both maximum strain and flexural strength. Notably, with the addition of 8 wt.% SF and 316L SS, despite the increase in the liquid phase, the presence of MgO and CaO from SF also increases. Excessive addition of MgO, due to the anchoring effect, inhibits the growth of ceramic grains [40]. In contrast, excessive CaO leads to the formation of a significant amount of CaO(Al_2_O_3_)_6_, which is deposited and anchors at grain boundaries, hindering the densification process of Al_2_O_3_ [41]. Moreover, excessive addition of SF and 316L SS leads to aggregation, creating stress concentration points and weak areas that become origins for crack nucleation and propagation [42], thus increasing the likelihood of intergranular fracture and reducing flexural strength.

Figure 14c summarizes the impact of SF and 316L SS content on the toughness of Al_2_O_3_. It is evident that adding SF and 316L SS effectively enhances the toughness of Al_2_O_3_, with the samples containing 6 wt.% SF and 316L SS exhibiting the highest toughness. Based on the analysis above, the overall performance of Al_2_O_3_ ceramics is optimal when the content of SF and 316L SS is 6 wt.%.

Though the results obtained from this work are promising, it is suggested that additional work be conducted. For example, this study specifically examines the effect of SF and 316L SS as second-phase particles to enhance Al_2_O_3_ ceramics. Future research should be conducted to investigate other aspects of property enhancement or to explore the impact of other materials on gradient Al_2_O_3_ for VPP printing. In this study, only four layers of Al_2_O_3_ ceramics were investigated, with SF and 316L SS ratios of 100%:0%, 66.7%:33.3%, 33.3%:66.7%, and 0%:100%. The enhancement of Al_2_O_3_ ceramics by additives with other layers and ratios will be explored in subsequent research.

## 4. Conclusions

This study explores the impact of varying contents of SF and 316L SS (0 wt.%, 2 wt.%, 4 wt.%, 6 wt., 8 wt.%) on the performance of gradient-printed Al_2_O_3_ parts using VPP technology. The main findings are as follows:

- A novel gradient printing process was proposed, distinguished from conventional VPP printing techniques by its method of creating green bodies with varying second-phase particle ratios across different layers.
- Experimental results indicate that Al_2_O_3_ parts printed through this process exhibit optimal performance when the content of SF and 316L SS is 6 wt.%, with shrinkage rates of 17.38%, 23.156%, and 19.316% in the X, Y, and Z directions, respectively, a porosity rate of 15.34%, and a water absorption rate of 5.935%.
- A more significant conclusion is that compared to Al_2_O_3_ parts without added SF and 316L SS, their flexural strength and toughness increased by 490.32% and 420.8%, respectively.
- This study provides implications for future research into the introduction of multiple second-phase particles for gradient printing of Al_2_O_3_ ceramics.

## Figures and Tables

**Table 1:** Chemical composition and element weight ratios of Al_2_O_3_ (provided by the vendor).

Grain Size	Element	Al_2_O_3_	Fe_2_O_3_	CaO	Na_2_O	TiO_2_	MgO	K_2_O	SiO_2_
100 nm	Wt.%	≥99.9	0.01	0.02	0.02	0.01	<0.01	0.01	<0.01
500 nm	Wt.%	99.9	0.0257	—	0.0153	0.0102	—	0.0086	0.0201

**Table 2:** Chemical composition and element weight ratios of 316L SS (provided by the vendor).

Element	Fe	Cr	Ni	Mo	Mn	Si	N	O	P	S	C
Wt.%	Balance	17	2.5	12	0.69	0.17	0.03	0.0226	0.005	0.0045	0.69

## Data Availability

The original contributions presented in the study are included in the article, further inquiries can be directed to the corresponding authors.
